# Synaptic modifications transform neural networks to function without oxygen

**DOI:** 10.1186/s12915-023-01518-0

**Published:** 2023-03-16

**Authors:** Lara Amaral-Silva, Joseph M. Santin

**Affiliations:** Division of Biological Sciences, The University of Missouri, Columbia, USA

**Keywords:** Hypoxia tolerance, Synaptic transmission, American bullfrog, Plasticity, Brain energetics

## Abstract

**Background:**

Neural circuit function is highly sensitive to energetic limitations. Much like mammals, brain activity in American bullfrogs quickly fails in hypoxia. However, after emergence from overwintering, circuits transform to function for approximately 30-fold longer without oxygen using only anaerobic glycolysis for fuel, a unique trait among vertebrates considering the high cost of network activity. Here, we assessed neuronal functions that normally limit network output and identified components that undergo energetic plasticity to increase robustness in hypoxia.

**Results:**

In control animals, oxygen deprivation depressed excitatory synaptic drive within native circuits, which decreased postsynaptic firing to cause network failure within minutes. Assessments of evoked and spontaneous synaptic transmission showed that hypoxia impairs synaptic communication at pre- and postsynaptic loci. However, control neurons maintained membrane potentials and a capacity for firing during hypoxia, indicating that those processes do not limit network activity. After overwintering, synaptic transmission persisted in hypoxia to sustain motor function for at least 2 h.

**Conclusions:**

Alterations that allow anaerobic metabolism to fuel synapses are critical for transforming a circuit to function without oxygen. Data from many vertebrate species indicate that anaerobic glycolysis cannot fuel active synapses due to the low ATP yield of this pathway. Thus, our results point to a unique strategy whereby synapses switch from oxidative to exclusively anaerobic glycolytic metabolism to preserve circuit function during prolonged energy limitations.

**Supplementary Information:**

The online version contains supplementary material available at 10.1186/s12915-023-01518-0.

## Background

Neural processing is energetically expensive in the vertebrate brain, which as a general rule, depends on continuous aerobic metabolism to fuel network function [1, 2]. Ion regulation following action potential firing and synaptic transmission are the most costly processes in the brain energy budget, being especially sensitive to oxygen deprivation [3–5]. It is traditionally accepted that the mammalian brain fails to maintain homeostasis during oxygen deprivation through deranged signaling at glutamatergic synapses. This leads to excessive Ca^2+^ and Na^+^ influx, causing hyperexcitability and depolarization [6, 7]. ATP produced by anaerobic glycolysis is insufficient to fuel the Na^+^/K^+^ pump, furthering the loss of the membrane potential and ion regulation [8–10]. Processes that buffer intracellular Ca^2+^ become saturated and the excess Ca^2+^,triggers mitochondrial apoptotic pathways, culminating in neuronal death [11, 12]. Hence, nearly all animals, including humans, cannot tolerate the lack of oxygen for more than a few minutes, and conditions that deprive the brain of oxygen are leading causes of death and long-term disabilities worldwide [13, 14].

Nevertheless, several animals live in hypoxic conditions or are submitted to bouts of hypoxia during their lives, such as during hibernation in borrows and ice-covered ponds, diving, flight at high altitudes, and living underground. These models have provided valuable insights for the development of new frameworks to prevent neural injury in humans during brain energy stress [15–17]. Many important mechanisms for neuroprotection were described in champion hypoxia-tolerant species such as western painted turtles, arctic ground squirrels, naked mole-rats, goldfishes, carp, and seals [18–21]. Although each species uses a distinct suite of mechanisms, most converge on the general principle of reducing neuronal activity to match the lower energy supply during hypoxia to maintain energy homeostasis through processes  referred as “synaptic,” “spike,” and “channel arrest” [22, 23].

American bullfrogs living in temperate zones hibernate in ice-covered ponds and, at this time, do not ventilate the lungs [24]. Thus, gas exchange occurs only through the skin, which decreases arterial oxygen tension (Po_2_) to <3 mmHg in some cases [25]. Although these values are exceptionally low, they are enough to support aerobic respiration due to reduced metabolic demands in the cold [25, 26]. However, in the spring, frogs need to emerge from hibernation by swimming to the surface to  takinge the first breath. For that, they need to restart several different motor and cognitive functions on the background of this very low P_O2_ that cannot normally fuel neural circuits. We have recently shown that motor networks in the frog brainstem crash after a few minutes of hypoxia. However, to overcome the challenge presented by emergence, these same networks transform to function for 20–30-fold longer without oxygen and glucose [27, 28]. These data support the view that the frog brain is normally “hypoxia-intolerant” but may have evolved plasticity mechanisms to resist the energetic stress encountered during emergence from hibernation.

To function during hypoxia and ischemia, our previous work showed that network activity in the frog brainstem shifts to rely solely on anaerobic glycolysis and internal glycogen breakdown [27]. These results are striking, as the ATP produced by anaerobic glycolysis—with or without glucose deprivation—has a limited capacity to maintain active synapses and action potential generation in diverse models and neuronal systems [29–36]. The transformation of an entire neural circuit to function during severe hypoxia suggests that synapses, firing, or both may improve their capacity to run on ATP from anaerobic glycolysis, a rare occurrence among vertebrates. Thus, we performed this study to determine the extent to which these cellular functions limit network output during hypoxia and then, by extension, which process undergoes energetic plasticity to fuel activity during hypoxia. To address this question, we used a novel semi-intact preparation that allows us to record motoneurons using patch-clamp electrophysiology while coupled to the respiratory rhythm-generating network [37]. Therefore, we could assess the endogenous activity of presynaptic circuits as postsynaptic currents on the motoneuron, as well as the postsynaptic motoneuron firing driven by these inputs. We further parsed the contributions of synaptic and cell intrinsic mechanisms in several reduced brain slice preparations.

## Results

**Fig. 1.**
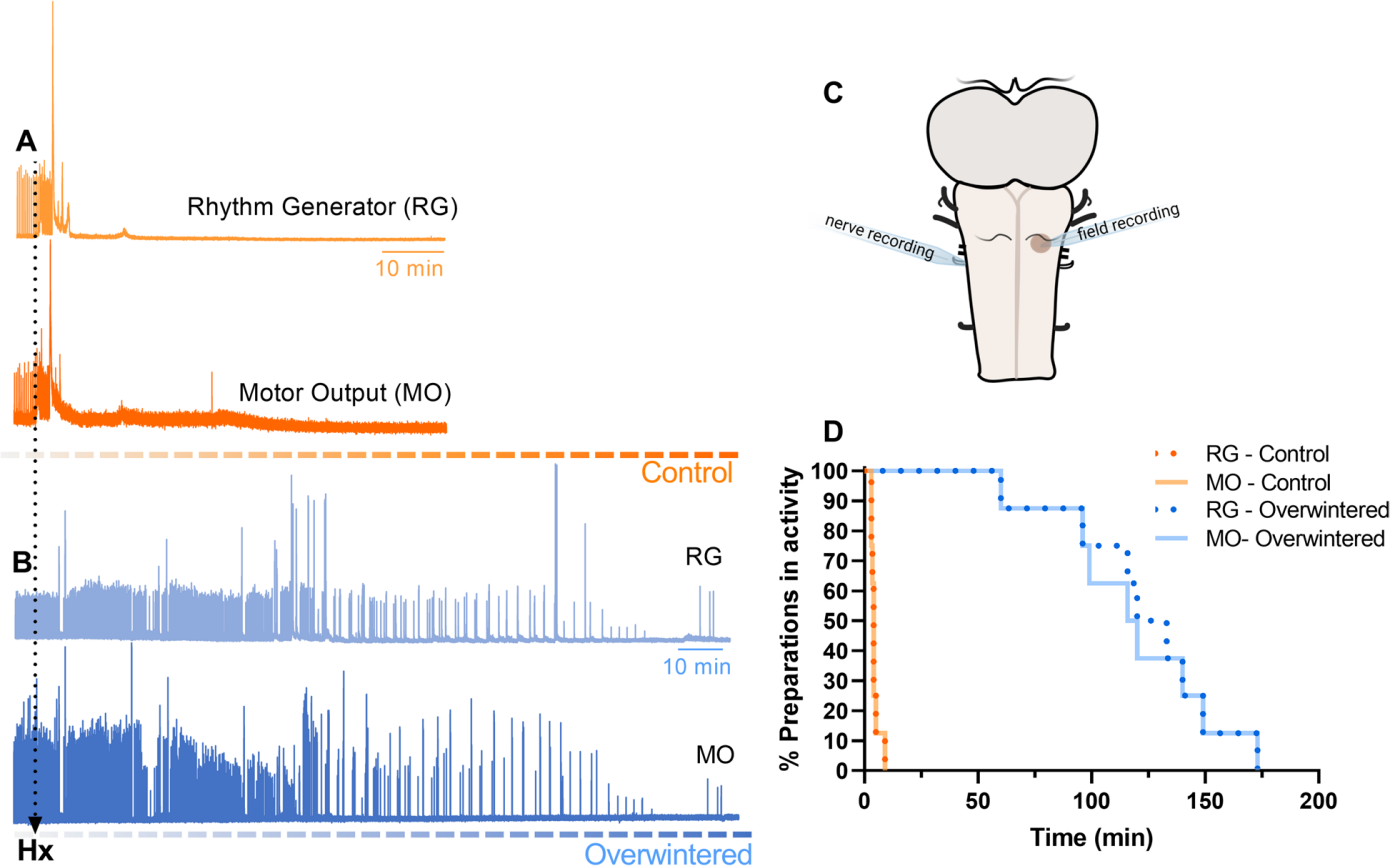
Overwintered frogs maintain brain function in hypoxia. Field recordings on the lung rhythm generator region (RG, top) were measured concurrently with the respiratory motor output on the vagus root (MO, bottom), as represented in the scheme (**C**). Individual raw traces exemplify the control (**A**, *n*=8) and overwintered (**B**, *n*=8) responses to hypoxia. **D** For control frogs, activity failed after a few minutes of hypoxia, differing from overwintered frogs that presented activity for hours in the absence of oxygen (*p* < 0.01). The output of the network and the rhythm generator region were not different within treatments (*p* > 0.05, Mantel-Cox test)

We previously showed that the frog respiratory network improves the duration of function from minutes to over 2 h during hypoxic exposure [27]. Here, we first replicated these experiments. In control frogs, motor nerve output associated with breathing (“fictive breathing”) failed after 4.1 ± 2 min of hypoxia, a period that increased by ~30-fold after overwintering (123 ± 32 min, *p* < 0.001). This phenomenon was observed both in the outflow from the vagal motor rootlet and local field potentials in the region of the respiratory rhythm generator for lung breathing [38, 39] (Fig. 1). These results corroborate our previous findings, showing dramatic functional improvements during hypoxia after emergence from overwintering.

**Fig. 2.**
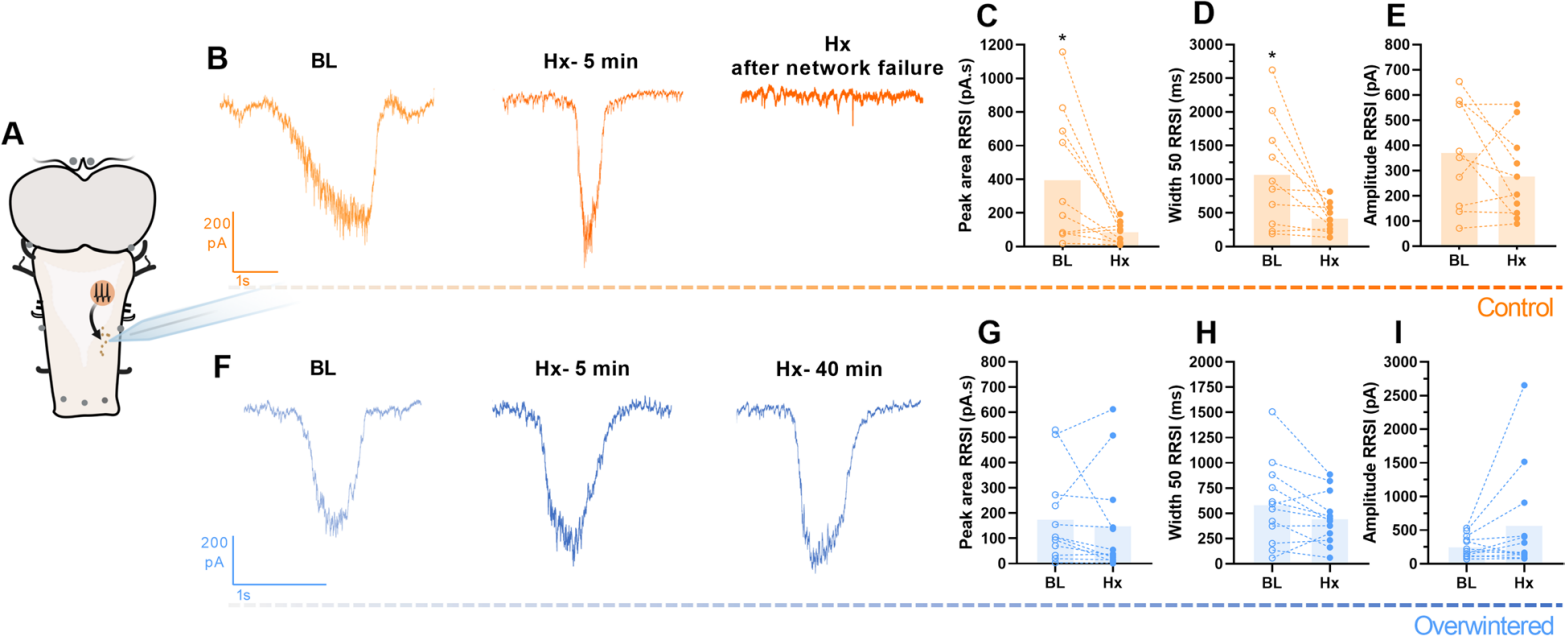
Brain transformation after overwintering avoids hypoxia disturbance on respiratory-related synaptic input (RRSI). Motoneurons receiving native input from the respiratory network (**A**) were recorded on control (*n*=10, top) and overwintered frogs (*n*=12, bottom) exposed to hypoxia. **B** The representative recording of a control neuron RRSI in baseline conditions (BL) is followed by the hypoxia effect on RRSI (Hx- 5 min) and the absence of RRSI concurrent to network failure (Hx after network failure). Controls’ RRSI in baseline conditions was compared to the last measurements before network failure (**C**–**E**). Upon hypoxia, controls presented a decrease in peak area (**C**) and width 50 (**D**) preceding network failure, while amplitude was not changed (**E**). **F** Representative recordings of an overwintered neuron RRSI in baseline conditions (BL) followed by the same period the hypoxia effect was recorded in controls (Hx- 5min) and after 40 min of hypoxia exposure (Hx- 40 min). In overwintered frogs, hypoxia exposure did not disturb peak area (**G**), width (**H**), and amplitude (**I**). Results were compared using paired *t*-test; *p* <0.05 was considered statistically significant (∗)

**Fig. 3.**
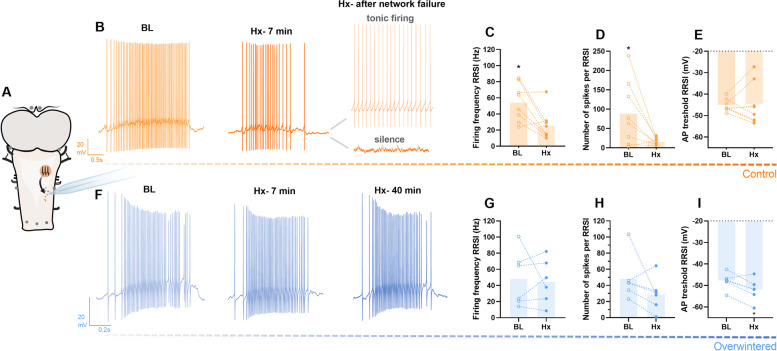
Respiratory-related firing is preserved in overwintered frogs despite hypoxia exposure. Firing of motoneurons receiving respiratory-related synaptic input (RRSI) from the rhythm generator regions (**A**) was recorded in controls (*n*=8, **B**) and overwintered frogs (*n*=6, **F**). **B** Representative recording of a control neuron firing in response to RRSI in baseline conditions (BL), when affected by hypoxia (Hx- 7min) and after network failure (Hx- after network failure), where both tonic firing and silence could be observed. Controls firing in baseline conditions was compared to the last measurements before network failure (**C**–**E**). Hypoxia exposure decreased control’s firing frequency (**C**) and number of spikes (**D**) triggered by the RRSI, with no change to the action potential (AP) threshold (**E**). **F** Representative recording of an overwintered motoneuron firing in baseline conditions (BL) followed by the same time that hypoxia affected firing in controls (Hx- 7min) and after 40 min in hypoxia (Hx- 40 min). Overwintering prevented the hypoxia effect on RRSI-triggered firing frequency (**G**) and number of spikes (**H**), as well as decreased AP threshold (**I**). Results were compared using paired *t*-tests, and a *p* < 0.05 was considered statistically significant (∗)

We next investigated the cellular processes that limit network activity, assessing synaptic transmission, and functions associated with a large burden on ion regulation (the membrane potential and action potential firing). In baseline conditions, there was no significant difference between any variable in control and overwintered animals (Additional file 1: Table S1). For control frogs, before network failure in hypoxia, the presynaptic input from rhythm-generating networks onto the motoneuron decreased, which was observed by a 77% reduction in the synaptic current area (*p* = 0.0230) and a 60% decrease in the width (*p* = 0.0265) (Fig. 2 top). The reduced synaptic drive was associated with a 52% decrease in firing frequency during respiratory bursts (*p* = 0.0136) and an 80% decrease in the number of spikes per burst (*p* = 0.0329, Fig. 3 top). After overwintering, however, motoneurons had no change in the respiratory-driven synaptic input for up to 40 min in hypoxia but trended towards increasing the synaptic current amplitude (122% rise, *p* = 0.1208, Fig. 2 bottom). In this way, the firing rate during the motor burst was maintained similarly to baseline values during hypoxia and presented a 9% decrease in the action potential threshold (*p* = 0.0281, Fig. 3 bottom), which may facilitate firing by reducing the difference between resting membrane potential and the threshold voltage. Motoneurons of cold frogs were analyzed at 40 min of hypoxia because it became difficult to maintain stable recordings after this time. However, in one extreme example, we maintained a stable voltage-clamp recording until network failure after 2 h of hypoxic exposure. The synaptic input associated with breathing was maintained throughout the entire period (Additional file 2: Fig. S1). Overall, these results point to a synaptic limitation on the network, which is overcome following overwintering.

**Fig. 4.**
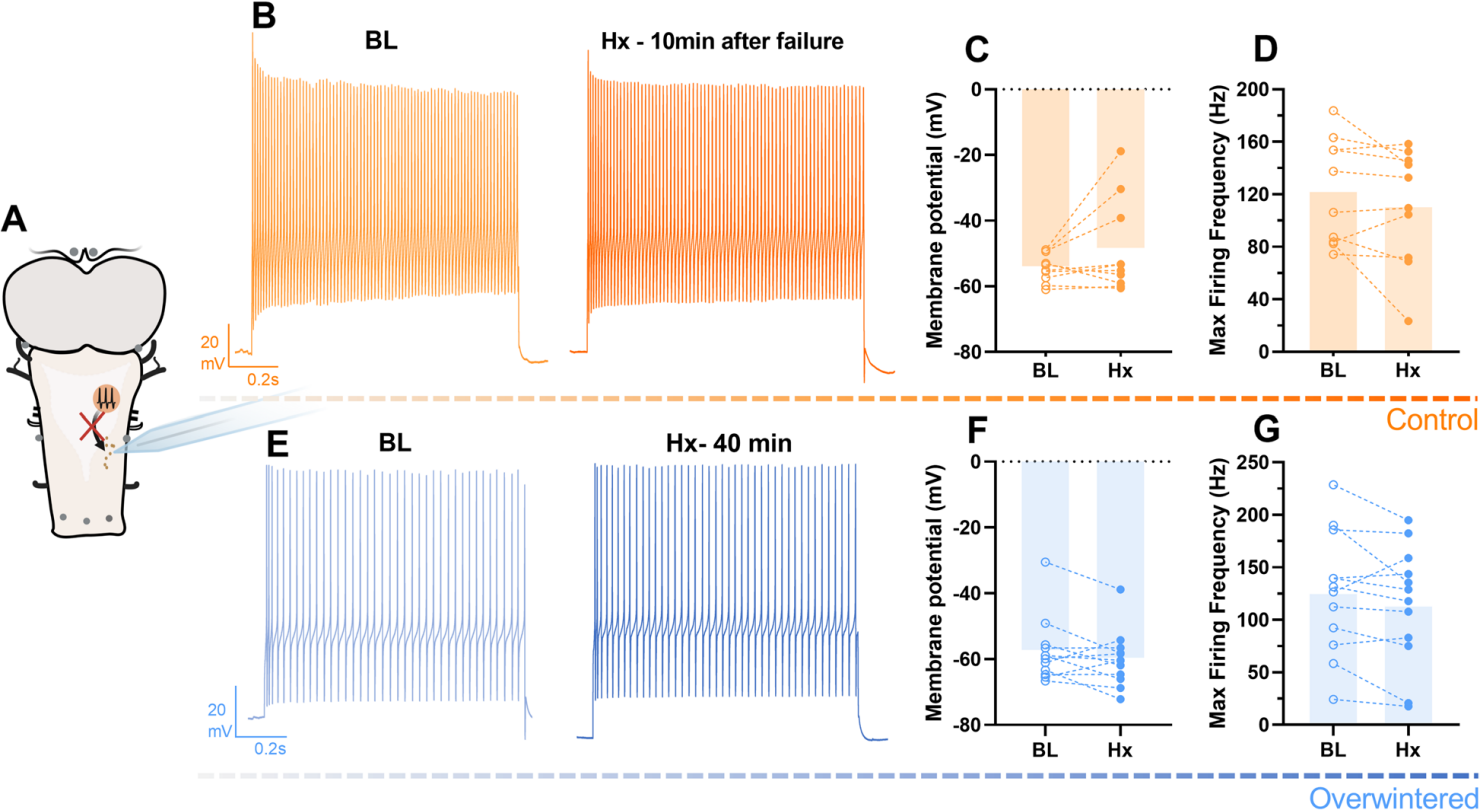
Membrane potential and firing frequency are preserved beyond network failure in hypoxia. Motoneurons in the semi-intact preparation (**A**) were analyzed regarding membrane potential and firing in response to a 1000-pA step current (max firing frequency). Recordings in baseline conditions (BL, left **B**/**F**) were compared to 10 min after the network output collapsed due to hypoxia in controls (*n*=10, right; **B**) or after 40 min of hypoxia exposure (active network) in overwintered frogs (*n*=12, right; **E**). This is exemplified by individual recordings in **B** and **E**. Hypoxia exposure did not change (*p*>0.05, paired *t*-test) membrane potential or max firing frequency in controls (**C** and **D**) or overwintered (**F** and **G**) motoneurons

**Fig. 5.**
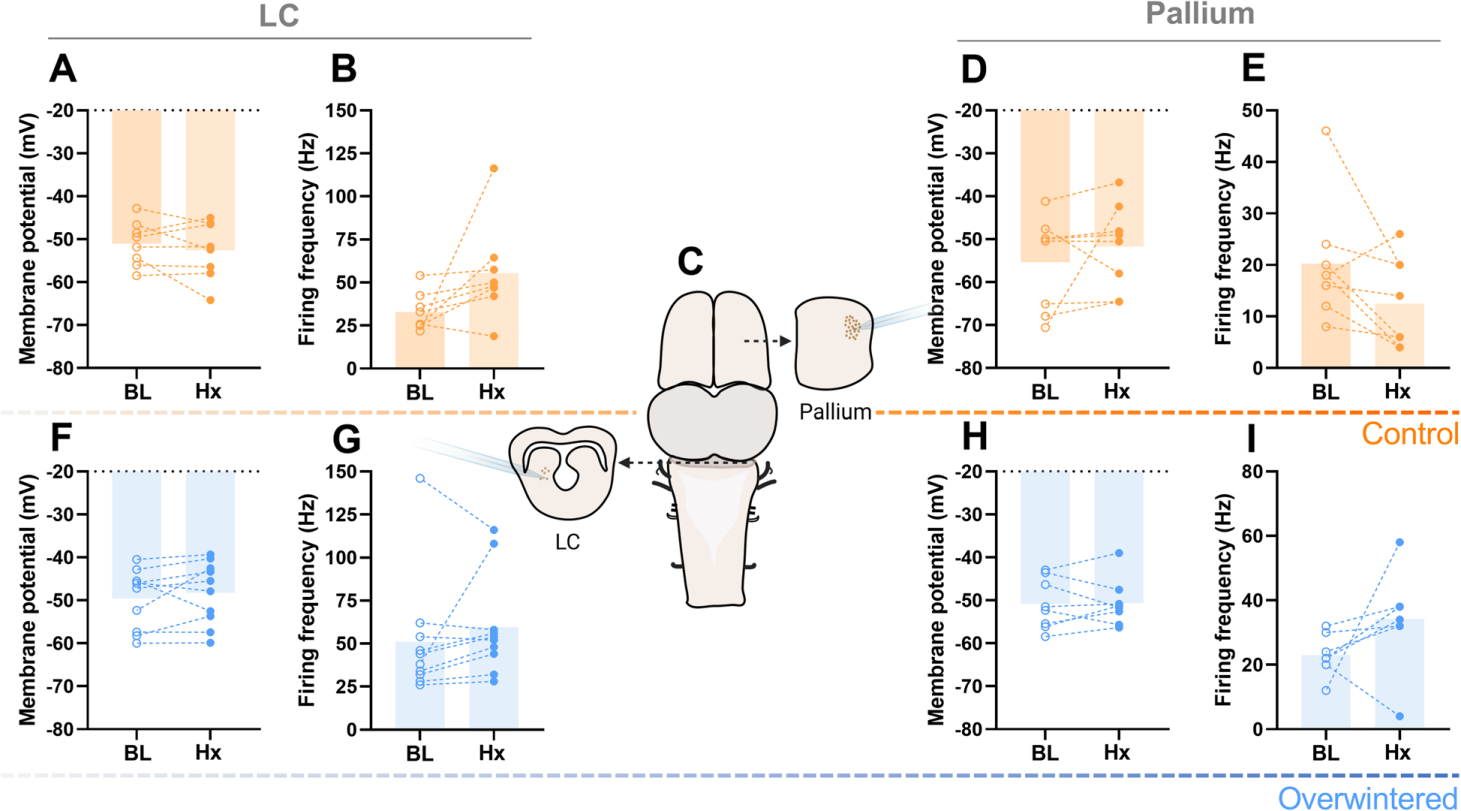
Membrane potential and firing frequency are not affected by hypoxia exposure throughout the brain. Neurons from locus coeruleus (LC) in brain slices (left, **C**) and from the pallium on a forebrain sheet dissection (right, **C**) were sampled from controls (LC *n*=8, pallium *n*=8, top) and overwintered (LC *n*=10, pallium *n*=8, bottom) frogs. Membrane potential and firing frequency in response to a 200-pA current step in LC cells and a 60-pA step in pallium cells during baseline conditions were compared  to 40 min of hypoxia exposure. There was no effect of hypoxia (*p*>0.05, paired *t*-test) in membrane potential and firing frequency in response to the current step both in LC and pallium neurons from controls (**A**, **B**—LC; **D**, **E**—pallium) or overwintered (**F**, **G**—LC; **H**, **I**—pallium) frogs

Although reduced synaptic drive led to reduced firing rates before failure in control networks, we noticed that membrane potential did not change for both groups and that control motoneurons often start firing tonically after the loss of respiratory synaptic input (Additional file 3: Fig. S2). Thus, we applied a step current and analyzed the firing rate in control motoneurons 10 min after network failure in hypoxia. To our surprise, motoneurons of control frogs were able to maintain membrane potential and firing frequency in response to the current injection after network failure (Fig. 4). Because the standard pipette solution for the whole-cell patch clamp includes 1 mM ATP, we tested if ATP supplied through the patch pipette could be fueling the motoneuron firing. This does not seem to be the case since a motoneuron recorded using the pipette solution with no ATP was also able to keep firing beyond network failure (Additional file 4: Fig. S3). In the mammalian brain, neurons in different regions present different tolerances to hypoxia, whereby caudal neurons seem more resistant and rostral neurons more susceptible to metabolic stress [40, 41]. Thus, to test if the “inexpensive” nature of firing was a regional vs. a global characteristic of the frog brain, neurons of the locus coeruleus (LC) and the dorsal pallium in the forebrain were recorded during hypoxia exposure. Intriguingly, we observed tonic firing in neurons from both regions throughout the hypoxic challenge, with no change in membrane potential or firing frequency in response to a current injection up to 40 min in hypoxia, which was similar in control and overwintered frogs (Fig. 5). In an additional experiment, we exposed a vagal motoneuron to hypoxia (1h) and evoked firing by injecting a step of current every 5 s to simulate continuous rhythmic inputs. This neuron was also able to maintain firing in hypoxia upon constant stimulation for the entire hour (Additional file 5: Fig. S4). In this way, processes that maintain the ion gradients to allow action potential firing seem to be upheld throughout the control frog brain during severe hypoxia.

**Fig. 6.**
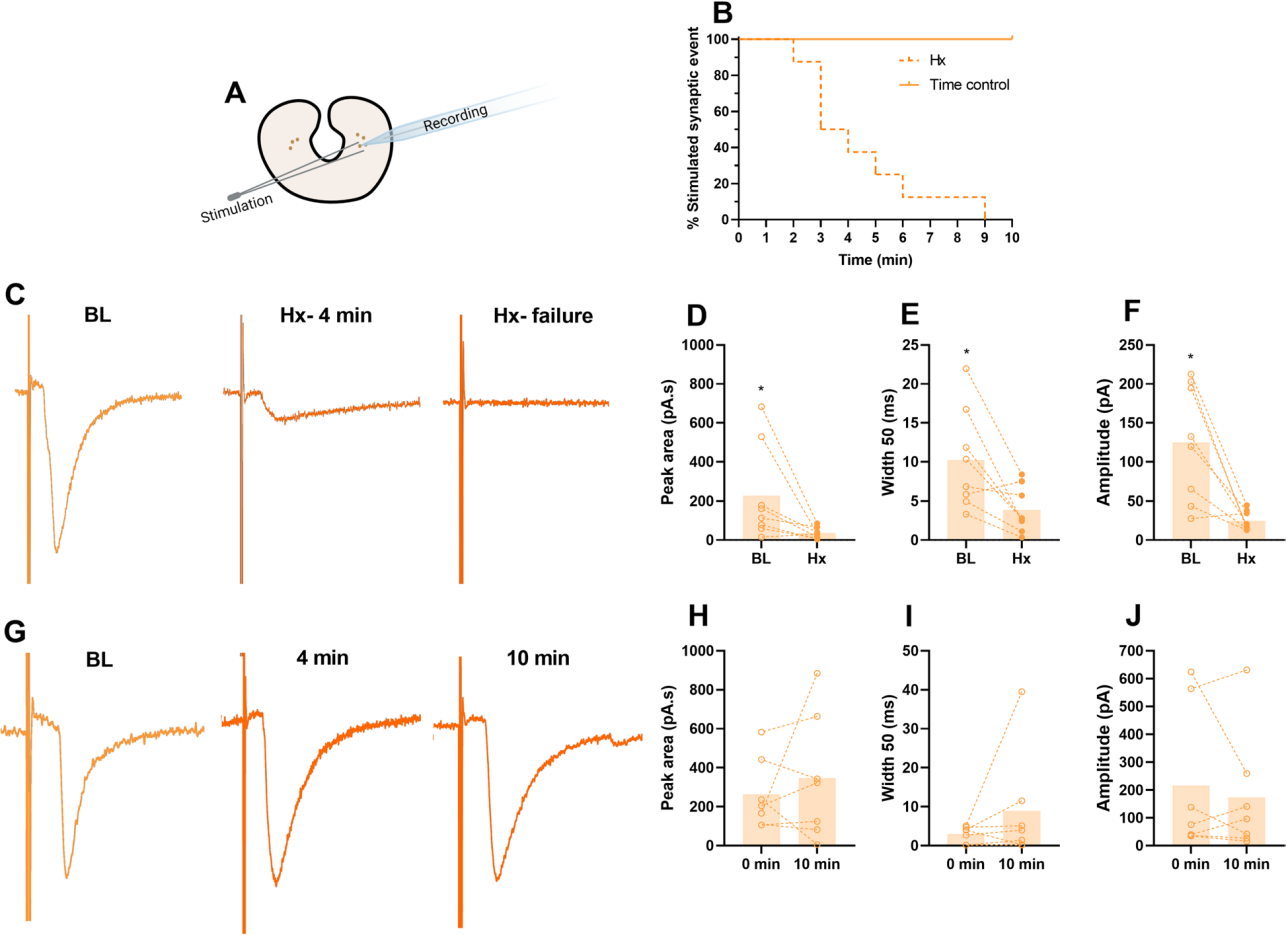
Evoked synaptic current is completely eliminated in control motoneurons exposed to hypoxia. Vagal motoneurons in a brain slice were recorded in a voltage clamp while nerve terminals were stimulated (**A**). No evoked synaptic events were observed after 10 min of hypoxia (*n*=8), while all time controls maintained activity throughout the whole period recorded (*n*=7, **B**). **C** Representative trace of a control neuron evoked synaptic current at baseline conditions (BL), when affected by hypoxia (Hx- 4min) and after failure in hypoxia (Hx- failure). Controls’ synaptic events in baseline conditions were compared to the events preceding failure in hypoxia. Hypoxia decreased the peak area (**D**), width (**E**), and amplitude (**F**) of the synaptic events before failure. **G** Representative recording of the evoked synaptic current in a time control motoneuron maintained in baseline conditions (BL), at the same time hypoxia affected current (4 min) and after 10 min of recording (10 min). When the motoneuron is maintained in baseline conditions, the peak area (**H**), width (**I**), and amplitude (**J**) of the evoked synaptic current are not affected. Results were compared using paired *t*-test; *p* <0.05 was considered statistically significant (∗)

**Fig. 7.**
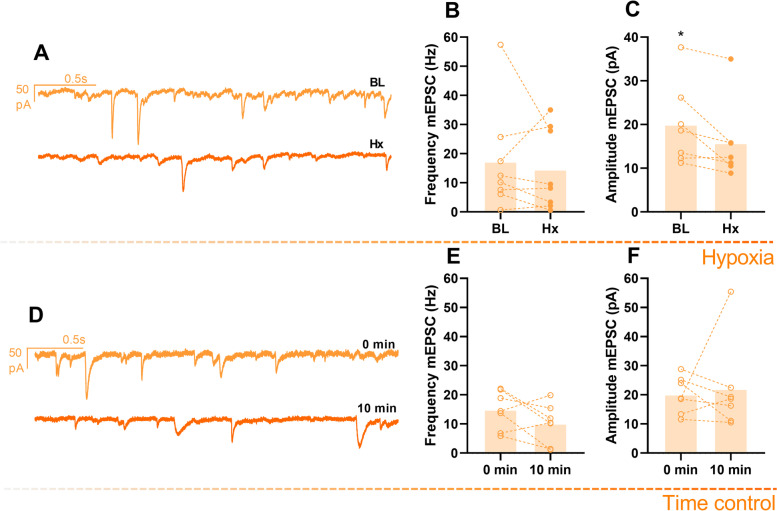
Miniature excitatory postsynaptic currents (mEPSCs) are reduced by hypoxia exposure in control motoneurons. **A** Representative traces of a motoneuron mEPSC recorded in slices before (top) and after hypoxia (bottom). Hypoxia did not affect frequency (**B**) but decreased amplitude (**C**) of the mEPSC (*n*=8). **D** Representative mEPSCs at 0- and 10-min recording in baseline conditions. Both frequency (**E**) and amplitude (F) were not affected in time controls (*n*=7). Results were compared using paired *t*-test; *p* <0.05 was considered statistically significant (∗)

Having ruled out the possibility that firing capacity limits network function in hypoxia, we then investigated the effect of hypoxia on evoked and spontaneous synaptic transmission directly. For that, we stimulated nerve terminals that innervate control motoneurons in slices and also assessed spontaneous quantal release events (miniature excitatory postsynaptic currents; mEPSCs) before and after hypoxia (Fig. 6). We evoked synaptic transmission using extracellular stimulation in the presence of TTX to isolate synaptic processes that do not involve generation of presynaptic action potentials, and bicuculline and strychnine to avoid inhibitory synaptic transmission. All evoked synaptic events failed within 10 min of oxygen deprivation. Before failure, these events had a decrease of 84% in the peak area (*p* = 0.048), 61% in width (*p* = 0.017), and 80% in amplitude (*p* = 0.006), which did not occur to time controls. Indeed, mEPSC had a 21% decrease in amplitude (*p* = 0.030) after hypoxia exposure, with no change in frequency (Fig. 7). Thus, we determined that synaptic failure, likely through pre- and postsynaptic mechanisms, is the limiting cellular process for network function that is overcome following hibernation.

## Discussion

The vertebrate brain has a high energy demand and is, therefore, especially susceptible to disruptions in oxygen delivery. However, we discovered that the bullfrog brain has a remarkable capacity to improve its function during oxygen deprivation upon emergence from hibernation [27], which we confirm in this study. During hypoxia, synaptic transmission weakens, leading to decreased motoneuron firing, ultimately causing the impairment of motor function in control frogs. Network output was not limited by the capacity to fire action potentials or maintain the resting membrane potential, but rather, through a combination of pre- and postsynaptic constraints. Overwintering, therefore, modifies excitatory synapses so that anaerobic glycolysis can fuel synaptic transmission during hypoxia to preserve network output.

### Functional hypoxia tolerance arises from changes in synaptic transmission but not action potential firing

Synaptic transmission and action potentials are recognized as cellular processes that consume most of the energy in the brain, relying on high rates of ATP synthesis [3, 4, 42, 43]. Contrary to our initial expectations, we observed that network failure in control frogs derives mainly from disruption of synaptic function since membrane potential is maintained and action potentials can still be fired with apparent ease during hypoxia. Despite traditional models of brain energetics that implicate action potentials as costly [3], our results support a number of studies pointing to synapses as the site with the highest energetic demands in a circuit. Direct measurements of ATP consumption in presynaptic terminals show that action potentials consume very little ATP, while synaptic vesicle release, recycling, and pH maintenance consume most ATP [4, 44–46]. Furthermore, synaptic failure occurs early in ischemia [31], and mild hypoxia may disturb synapses but not action potential firing [47]. Indeed, in a penumbra model, synaptic connections weaken within the first hours of hypoxia and completely dissemble after 48h despite the maintenance of firing activity and action potential properties throughout the energetic insult [48]. Thus, synapses seem to be generally the first circuit process affected by a lack of oxygen supply, and modulation of synaptic energetics is a key factor for network function during hypoxia.

The maintenance of the membrane potential and the capacity for action potential firing in hypoxia was not a specialized feature of brainstem motoneurons but occurred in neurons from different regions across the brain. Indeed, firing was maintained after 40 min of hypoxia in neurons of the LC, a midbrain structure involved in the control of breathing and arousal [49, 50], and neurons of the pallium, a forebrain structure homologous to the dorsal cortex/Wulst of sauropsids and the mammalian neocortex [51]. These results suggest that action potential firing in frogs is generally inexpensive relative to mammals and that frogs do not exhibit the rostrocaudal gradient in hypoxia tolerance was observed in mammals, where brainstem neurons survive energetic challenges that kill cortical neurons [40, 41].

During hypoxia, neurons from various species die through a process termed “anoxic depolarization,” which involves over-stimulation of glutamate receptors, excessive depolarization, and a failure to regulate ion concentrations due to a plummeting ATP reserve [10, 52–56]. Thus, the maintenance of membrane potential under hypoxia across the frog brain shows an impressive ability to avoid anoxic depolarization and excitotoxic cell death. Our data corroborate experiments in *Rana pipiens* demonstrating extracellular glutamate increases (indicating cell depolarization) only after 1.5 h after the ATP depletion [57]. In fact, strategies to minimize anoxic depolarization are used for neuroprotection in hypoxia-tolerant goldfishes, turtles, and seals, which are able to present milder depolarization [58–60]. Here, we show that amphibians are able to maintain membrane potential in hypoxia similar to baseline values and fire action potentials with relative ease during severe hypoxia in both groups, even in control animals. These results further support our conclusion that synapses represent the main cause of improved function during energy stress after overwintering.

### Frogs’ functional hypoxia tolerance differs from strategies used by champion hypoxia-tolerant species

Hypoxia-tolerant brains have been long proposed to reduce metabolic demands to survive the lack of oxygen using mechanisms such as channel arrest [23]. Indeed, animals such as the western painted turtle reduce the membrane conductance to Na^+^ and K^+^ ions and arrest AMPA and NMDA receptors while increasing GABAergic transmission to dampen excitability [61–66]. Each of these mechanisms aims to arrest spiking, thus  reducing the energy demand during hypoxia by minimizing neuronal activity. Accordingly, a synaptic arrest hypothesis has been proposed to explain the full extent of the decrease in neuronal activity to match low fuel in hypoxia [22]. Similar strategies are also observed in other hypoxia-tolerant species, such as goldfish, which also launch spike and channel arrest in response to hypoxia [59, 67]. Hypoxia-tolerant arctic ground squirrels have lower expression of subunit 1 of NMDA-glutamate receptors and reduced NMDA receptor-mediated Ca^2+^ influx following glutamate application compared to mice [21]. In addition, deep-diving hooded seals reduce the amplitude of field EPSPs to ~30% of baseline values to maintain synaptic activity for at least 3h of hypoxia [18]. A similar pattern seems to occur in naked mole-rats that also decreases glutamate during hypoxia [19, 68]. Thus, for most animals that endure hypoxia, spike and synaptic arrest appear to be a common strategy.

In contrast, to emerge from hibernation, frogs must restart adaptive behaviors, such as the neural circuits that produce breathing, on the background of critically low Po_2_ values [25]. In this scenario, arrest strategies are clearly not an option. Unlikely species that are always hypoxia tolerant, such as the ones described above, we show that frogs dramatically improve circuit function in hypoxia compared to their control condition by maintaining synaptic transmission. Thus, functional hypoxia tolerance, as we describe in overwintered frogs, defies the premise that decreasing synaptic activity to match low energy production is the key to withstanding hypoxia [69]. Is this ability to maintain neuronal function in hypoxia unique to the frog? Although synaptic arrest mechanisms work well for other species, we speculate that certain environmental challenges may require behaviors in the presence of low Po_2_, providing a strong pressure to switch from an “arrest strategy” to a functional strategy. For example, turtles may be vigilant and process sensory information during hibernation in hypoxic water [70]. In any case, future studies should address parallels and differences in hypoxia tolerance mechanisms throughout the brain and in response to environmental challenges across species.

### Synaptic adjustments for hypoxia tolerance in overwintered frogs

After overwintering, frogs were able to maintain synaptic function in hypoxia, which sustained motoneuron firing and allowed for network activity to continue. Despite no difference in the baseline values between control and overwintered animals (Additional file 1: Table S1), overwintered frogs seem to present decreases on average in synaptic variables (respiratory-related synaptic input peak area and width 50 were ~2× and ~1.8× smaller in overwintered frogs compared to controls). This might serve to save energy after overwintering, aiding in keeping network function in hypoxia. In addition, emergence from hibernation is known to change synaptic processes [71]. We have shown that frogs have greater miniature excitatory postsynaptic currents in vagus motoneurons after overwintering, which points to increases in AMPA receptors [72, 73]. We speculate that increases in postsynaptic receptors may allow presynaptic neurotransmitter release to decrease while causing a similar network-driven synaptic current (Fig. 2), lowering the cost for the most expensive component of synaptic transmission [4, 44–46]. Nevertheless, when the preparation is exposed to hypoxia, maintenance of function seems to be prioritized by synaptic and intrinsic mechanisms. The width and peak area of synaptic input were maintained, and amplitude slightly increased after 40 min of hypoxia. This was combined with a decreased action potential threshold during hypoxia, which would allow less synaptic input to bring the neuron to fire. Firing at lower voltages would both facilitate network activity and potentially use less energy to recover postsynaptic ion gradients. Indeed, some neurons seem to spend more ATP on recovering the ionic disturbances from postsynaptic currents than on action potentials [46]. However, the specific mechanisms that change to allow for the maintenance of synaptic function in overwintered frogs remain to be studied.

We previously described that overwintered frogs maintain network function without aerobic respiration by blocking the electron transport chain with cyanide during severe hypoxia. However, the network stops following the inhibition of glycolysis by iodoacetate, demonstrating glycolytic dependence for network activity [27]. Since the synaptic function is transformed after overwintering, its demands (vesicle recycling, calcium homeostasis, vesicle pH regulation, pre/postsynaptic ion regulation) must be met without ATP synthesis from aerobic metabolism and, thus, fueled only by anaerobic glycolysis [27]. This is no small feat since synaptic terminals consume large amounts of ATP [44–46]. For example, to supply the resting demand of a synaptic terminal in *Drosophila*, ATP must be produced at a rate of 52 nmol min^−1^ μl^−1^, which increases to 963 nmol min^−1^ μl^−1^ during activity. The ATP required for both maintenance and active transmission is almost entirely supplied by mitochondrial aerobic metabolism, while glycolysis contributes only ~0.4% [34]. Although glycolytic enzymes may translocate to presynaptic terminals to support motor behavior for a few minutes during anoxia [74], it is not clear how synapses change to function for up to 2 h (Additional file 2: Fig. S1) using only anaerobic glycolysis. Nevertheless, recent data from our lab suggests that a transcriptional program promotes glycolysis and deemphasizes aerobic metabolism after overwintering [75], pointing to changes in the energy supply. Even though we observed an increase of co-expression in genes for glucose metabolism and glycogenolysis  in this study, at this point, there is still a possibility of fructose-driven glycolysis to resist hypoxia after overwintering as it is described in naked mole-rats [76]; thus, further studies are needed to clarify the substrate(s) that power glycolysis in this condition. Additionally, we speculate that multiple aspects of synaptic transmission may enhance their efficiency to reduce the amount of ATP needed to maintain similar performance, decreasing demand. Guided by the present results, exciting future work will address mechanisms that allow synapses to switch their fuel to only anaerobic glycolysis while undergoing a massive functional improvement in the absence of aerobic metabolism.

### Perspectives

Evolutionary hypotheses for brain energetics assert that natural selection has optimized the efficiency of information transfer at synapses based on the ATP supplied by aerobic respiration [77, 78]. Yet, we suggest that some circuits may retain a large capacity to improve synaptic efficiency to the demands of the animal. Amphibian brainstem synapses appear to fundamentally change their energetic support system to function in hypoxia after overwintering, indicating they normally exist far from their “optimal” efficiency in summer and fall seasons. Defining the environmental cues, integration pathways, and effector responses that alter synapses to function during energy depletion promises unique insights to understand brain energetics in health and may aid in overcoming energetic stress in diseases that converge on impaired oxygen supply and mitochondrial defects at synapses (Campbell et al., 2019; Feigin et al., 2022; Li and Sheng, 2022).

## Material and methods

### Animals

All experimental procedures were approved by the Institutional Animal Care and Use Committee at The University of North Carolina at Greensboro (protocol #19-006). Adult American bullfrogs (*Lithobates catesbieanus*) with no sex distinction weighing 100±15 g were acquired from Rana Ranch (Twin Falls, ID, USA) and housed in plastic tanks containing dechlorinated and aerated water in a 12/12 light/dark cycle. The animals were then randomly assigned to a control or an overwintering group, where hibernation conditions were simulated in the laboratory. Frogs in the control group were acclimated to the laboratory conditions for at least a week before the experiments. In this period, they were maintained at room temperature (23±2°C) with access to wet and dry areas and were fed with pelleted food provided by Rana Ranch once a week. Frogs in the overwintering group were placed in controlled temperature incubators (Thermo Fisher Scientific, Waltham, MA, USA), where the temperature was gradually reduced to 4°C over 10 days. Once at 4°C, screens were placed directly below the water level to impede access to the surface, and they were kept in this environment for 30 days before being used in experiments. Since the frogs do not eat in cold temperatures, overwintered animals were not fed. For the experiments, overwintered frogs were acclimated at room temperature for ~20 min before decapitation, and all the analyses proceeded at 22±2°C.

### Dissection

The frogs were deeply anesthetized using 1 mL of isoflurane in a sealed container until loss of the toe-pinch reflex. They were then decapitated, and the head was immersed in the artificial cerebral spinal fluid (aCSF, composition in mM: 104 NaCl, 4 KCl, 1.4 MgCl_2_, 7.5 d-glucose, 1 NaH_2_PO_4_, 40 NaHCO_3_, 2.5 CaCl_2_, all purchased from Fischer Scientific, Waltham, MA, USA). This was bubbled with 1.5% CO_2_ and 98.5% O_2_ to oxygenate the brain while matching bullfrog arterial pH of ~7.85 at ~20°C [79, 80]. Decerebration was immediately performed, and the brainstem-spinal cord was carefully removed as described previously [37, 72, 81].

### Tissue preparations

This study used several tissue preparations: intact, semi-intact, and brain slices. Below, we describe procedures for generating each type of preparation.

#### Motoneuron labeling

For all preparations involving motoneuron recordings, we labeled cell bodies for unambiguous identification of this cell type. Following dissection, the 4th branch of the vagus nerve complex was labeled bilaterally using a fluorescent dye (tetramethylrhodamine dextran 3000 MW; Invitrogen-Thermo Fisher, Waltham, MA, USA). This branch contains axons that mostly innervate the glottal dilator muscle, which is involved in lung ventilation in anuran amphibians [82, 83]. The nerve was pulled into a fire-polished pipette, and ~1μL of tetramethylrhodamine dextran 3000 MW was injected into the tip of the pipette in contact with the nerve for 1h on the first side and subsequently for 2h in opposite as previously described [27, 37, 72].

#### Semi-intact preparation

After labeling, the brainstem was horizontally sliced in a semi-intact preparation where only the vagal motoneurons are exposed, keeping the synaptic inputs of the native respiratory network. This preparation design and its output were previously described in detail [37]. Briefly, the brainstem was attached by agarose to an agar block keeping the most rostral side at a 45° angle, preventing it from being sliced. The vagal root was positioned over the angled edge (between 45 and 0°), maintaining the brainstem’s caudal part over the agar block’s horizontal side. This part of the brainstem was covered with agarose, and the block was glued to the vibratome plate (Technical Products International series 1000, St. Louis, MO, USA), where it was immediately immersed in oxygenated 4°C aCSF. In sequence, the dorsal part of the caudal brainstem was sliced at 200 μm until the bottom of the 4th ventricle was approached in the region between vagal and hypoglossal nerves. Then, 10- to 50-μm slices were cut to expose the region containing vagal motoneuron cell bodies, while the region containing neurons responsible for the lung rhythm generator was preserved [38, 84, 85]. The semi-intact preparation was pinned in a Sylgard-coated chamber for electrophysiology recordings (RC26G, Warner Instruments Holliston, MA, USA) and left to recover for 1 h before the recordings. In this period, the preparations were continuously superfused (~7mL/min) with aerated aCSF (98.5% O_2_ and1.5% CO_2_) using peristaltic pumps (Watson Marlow, Falmouth, CNL, UK) at room temperature (21°C±1).

#### Slices

In other experiments, the brainstem was sliced in cross sections to access labeled vagal motoneurons without rhythmic presynaptic input and neurons of the locus coeruleus (LC) [50, 86, 87]. For that, after dissection, the ventral side of the brainstem was glued to an agar block using super glue. The agar block was glued to the vibratome plate, and the vagal motoneurons were exposed by transversally slicing the vagal motor pool area at 300 μM (Burton and Santin, 2020; Santin et al., 2017; Zubov et al., 2021; Zubov et al., 2022). The midbrain was sliced at 400μM to access LC neurons [50, 87]. Additionally, the forebrain was dissected to access neurons of the pallium, the vertebrate precursor of the cortex [51, 88]. The pallium was dissected using the same method applied to access turtle cortical cells [89]. Briefly, after dissection, the forebrain was cut using an ophthalmic scissor dorsal to the lateral amygdala separating the pallium from the ventral regions. The pallium was then opened as a sheet, and dorsal pallium cells were used for the experiments [51].

### Electrophysiology

#### Whole brainstem—extracellular motor root and field potential recordings

The freshly dissected brainstem-spinal cord was pinned with the ventral side up in Sylgard (Dow Inc. Midland, MI, USA)-coated 6-mL Petri dishes where it was continuously superfused (~7mL/min) with aerated aCSF (98.5% O_2_ and 1.5% CO_2_) using peristaltic pumps (Watson Marlow, Falmouth, CNL, UK). All preparations were recorded at room temperature ~22°C. Borosilicate glass pipettes were pulled (Sutter Instruments, Novato, CA, USA) and manually adjusted in two sizes using sandpaper and fire polishing. A bigger size was used to ensure a tight seal around the vagal nerve root, and a smaller size was used to record from the lung generator cells. Rhythmic lung activity was recorded by an electrode placed in the area identified as the bullfrog’s lung rhythm generator [38, 39]. Extracellular signals from both regions were amplified (×1000) and filtered (low pass, 1000 Hz; high pass, 100 Hz) using an AM-Systems 1700 amplifier (Sequim, WA, USA). The signal was digitized using Powerlab 8/35 (ADInstruments, Dunedin, Otago, New Zealand), rectified, and integrated (100 ms τ) using the LabChart data acquisition system (ADInstruments, Dunedin, Otago, New Zealand).

#### Semi-intact preparation

The preparation was pinned in a Sylgard-coated recording chamber and transferred to the patch clamp set-up, where it was superfused with aCSF by gravity at a ~1–2-mL/min rate. The vagal nerve root was firstly identified at 4× magnification using a real-time imaging camera (Hamamatsu ORCA Flash 4.0LT sCMOS, Hamamatsu Photonics, Hamamatsu, SZ, Japan) and pulled into a fire-polished glass pipette suction electrode for extracellular recordings. After obtaining stable extracellular recordings to monitor network activity, labeled motoneurons were identified and used for patch-clamp experiments. The neurons were identified at 40× magnification and approached by glass pipettes (2–4 MΩ resistance) filled with a solution containing (in mM) 110-K-gluconate, 2 MgCl2, 10 HEPES, 1 Na2-ATP, 0.1 Na2-GTP, and 2.5 EGTA. The pipette was attached to a head stage (CV203BU) connected to an MP-285 micromanipulator and an MPC-200 controller (all Sutter Instruments, Novato, CA, USA). Positive pressure was applied to the tip of the pipette while approaching the cell and quickly removed, gentle negative pressure was used to form a >1GΩ, and the whole-cell access was obtained by breaking the seal with rapid negative pressure. After assuring stable access in the cell, voltage clamp was performed to verify the respiratory-related synaptic inputs (RRSIs) in neurons clamped at −66 mV. Membrane potential and respiratory-related firing behavior were measured in the current clamp. Firing ability was determined by a step protocol where the firing frequency-current (*F*-*I*) relationship was analyzed by injecting −150 to 1000 pA of current. All data were acquired in pClamp 11 software using Axopatch 200B amplifier and Axon Digidata 1550B digitizer (all from Molecular Devices, San Jose, CA, USA).

#### LC and pallium neurons

Slices containing LC cells and the pallium sheet were transferred to the recording chamber, stabilized using a nylon grid, and bathed with aCSF fed by gravity. After identifying and patching the cells as described above, membrane potential was measured in the current clamp, and the F-I relationship was determined in a step protocol. For LC neurons, the current injected for 0.5s ranged from −150 to 500 pA, and for pallium cells, −20 to 80 pA or until the cell achieves depolarization block.

#### Motoneurons in slices—evoked synaptic transmission

Slices containing labeled vagus motoneurons were transferred to a recording chamber and stabilized by a nylon grid. The motoneurons were identified (40×), and a bipolar tungsten stimulation electrode with tip separation of 250 μm (MicroProbes, Gaithersburg, MD, USA) was positioned ~120μm from the cell soma. The cell was then patched as described above and monitored in a voltage clamp, hold at −80mV. Nerve terminals in the slice were stimulated by 20-μs pulses every 2 s by an AM-Systems constant-voltage isolated stimulator (Sequim, WA, USA). The stimulus intensity was adjusted to produce a reliable synaptic response with the minimum voltage, which was monitored and adjusted if necessary for 2 min before starting experimental protocols to ensure the evoked current was stable.

### Experimental procedures

#### Whole brainstem—extracellular motor root and field potential recordings

The output of whole brainstem preparation was monitored for 4h after decapitation to ensure a stable baseline for all recordings. Fictive breathing and activity of the lung generator were then recorded in baseline conditions for 10 min and throughout 4 h of hypoxia exposure, both for the controls and overwintered frogs.

#### Semi-intact preparation recordings

Motoneurons of controls and overwintered frogs had initial voltage and current measurements in baseline conditions (98.5% O_2_, 1.5% CO_2_) and, in sequence, were submitted to severe hypoxia, where aCSF was bubbled with 98.5% of N_2_ and 1.5% of CO_2_. Using this anoxic gas mixture, we previously observed ~2% of oxygen in the bath [27], which should result in tissue anoxia since the Po_2_ in the frog brainstem preparation has P_O2_ ~0 kPa near the brain surface and within the brain tissue at any depth when aCSF is gassed with a similar gas mixture [90]. While in hypoxia, voltage and current clamp protocols were alternated every 2 min. To investigate which cellular component was limiting network function, we compared the last measurements before network failure in control animals to 40 min of hypoxia in overwintered preparations in which all preparations still produced network activity. We used this approach because the respiratory network of control animals typically failed after a few minutes of hypoxia, and preparations of overwintered frogs keep working for several hours [27]. The time recorded in the motoneurons of overwintered preparations was limited by the inherent difficulty of maintaining long-term patch-clamp recordings in this preparation; 40 min represented the length of time that could be achieved in most neurons. The recording quality seemed to decrease over time only due to a technical limitation associated with maintaining quality patch seals and not as an effect of hypoxia since we could patch cells when the tissue was still hypoxic after the 40 min recording period (data not shown). Indeed, in an extreme example, we recorded one cell for the whole time until network failure after 2h of hypoxia exposure (Additional file 2: Fig. S1). Step currents were performed once in baseline conditions and once 10 min after the network failure in hypoxia for control animals. For overwintered animals, the second step proceeded after 40 min of hypoxia.

#### LC and pallium neurons

LC and pallium neurons had membrane potential verified, and step currents were injected in baseline conditions, which was followed by hypoxia exposure. Neurons were monitored in voltage and current clamp, and after 40 min of hypoxia, membrane potential and firing ability were analyzed again. Control and overwintered frogs were used in those experiments.

#### Motoneurons in slices—evoked synaptic transmission

To evaluate the hypoxia effect in the synaptic input per se, presynaptic terminals of vagal motoneurons isolated in slices were stimulated in baseline conditions. After assuring stable initial recordings in the voltage clamp, the slice was bathed with oxygenated aCSF containing 200nM TTX, 20 μM bicuculline, and 2 μM of strychnine to ensure that the evoked transmission had no influence from action potentials or inhibitory synaptic transmission. This solution was washed in for 2 min before the current was injected by the stimulator. After a stable current injection, the cell was recorded in voltage clamp while (a) perfused with aCSF+inhibitors, bubbled with 0% O_2_, and (b) monitored in an oxygenated solution for time control. Five stimulated postsynaptic currents (sEPSC) in baseline conditions were compared to the 5 last events in hypoxia or at 10 min of recording in time controls. Miniature excitatory postsynaptic current (mEPSC) was analyzed between stimuli at the same period sEPSC was analyzed.

### Data analysis

Respiratory-related synaptic inputs, evoked synaptic current, and mEPSCs were recorded in voltage clamp, and their amplitude, width 50, peak area, and frequency were analyzed using the peak analysis function of LabChart (ADInstruments, Dunedin, Otago, New Zealand). The recordings were checked by eye to ensure the accuracy of the program in identifying the events. For mEPSC, a cutoff of 7pA was applied in amplitude. Input resistance was calculated using Ohm’s law (*R*=Δ*V*/Δ*I*) after recording the changes in voltage due to −100-pA current injection and was used to determine access quality. Membrane potential was determined in the current clamp in the period between respiratory bursts. The action potential threshold was considered at the last point before the action potential was triggered naturally by respiratory synaptic input. Firing frequency and the number of spikes triggered by RRSI included all spikes during a single event, which was analyzed for all events in a minute of current-clamp recording. Firing frequency in response to current was analyzed upon injection of 1000pA in vagal motoneurons (or until depolarization block), 200pA in LC neurons, and 60pA in pallium cells. Firing frequency in response to RRSI or current was calculated using the frequency function in the cyclic measurements function on LabChart (ADInstruments, Dunedin, Otago, New Zealand).

### Statistical analysis

Data are raw values from individual experiments accompanied by mean. The hypoxic effect on RRSI’s and sEPSC’s peak area, width 50, and amplitude, as well as membrane potential, firing frequency, and mEPSC’s amplitude and frequency, were analyzed using paired *t*-test for both control and overwintered animals. Differences in activity time in the network/lung area and sEPSC were examined in a survival curve by the log-rank Mantel-Cox test. Statistical significance was accepted when *p ≤ 0.05*. Baseline values of control and overwintered frogs were compared using an unpaired *t*-test, and Welch’s correction was applied in case of different standard deviations. The number of cells and preparations (*n*) used for each experiment is indicated in the figures.

## Supplementary Information

**Additional file 1: Table S1.** Baseline values of physiological variables measured in control and overwintered frogs.

**Additional file 2: Figure S1.** Respiratory-related synaptic input recorded in an overwintered motoneuron for 2 hours in hypoxia.

**Additional file 3: Figure S2.** Concurrent recording of vagal motoneuron action potentials and respiratory network output of a control frog during hypoxia-induced network failure.

**Additional file 4: Figure S3.** Recording of a control vagal motoneuron firing in response to a 1000pA step current in baseline conditions and after 10 minutes of network failure using a pipette solution with no ATP.

**Additional file 5: Figure S4.** Recording of a control motoneuron showing that it does not change firing in response to intermittent current injection up to 1 hour of hypoxia exposure.

## Data Availability

All data generated or analyzed during this study are included as individual values in the figures within this published article and its supplementary information files. Raw data is available in the additional files and from the authors.

## Abbreviations

Po_2_: Arterial oxygen tension
LC: Locus coeruleus
mEPSCs: Miniature excitatory postsynaptic currents
RRSI: Respiratory-related synaptic input
aCSF: Artificial cerebral spinal fluid
sEPSC: Stimulated postsynaptic currents
RG: Lung rhythm generator region
MO: Motor output
BL: Baseline conditions
Hx: Hypoxia
AP: Action potential
