# Supplementary material for: Synaptic modifications transform neural networks to function without oxygen
Source: BMC Biol. 2023 Mar 16;21:54. doi: 10.1186/s12915-023-01518-0 (PMC10022038; doi:10.1186/s12915-023-01518-0)
Supplement: Supplementary file 1 — Additional file 1: Table S1. Baseline values of physiological variables measured in control and overwintered frogs. [file 12915_2023_1518_MOESM1_ESM.pdf]

SUPPLEMENTAL TABLE

Additional file 1: Table S1. Baseline values of physiological variables measured in control and overwintered frogs

Variable	Control	Overwintered	p value	Cell type / preparation	Figure
Peak Area RRSI (pA.s)	400.3±394.5(10)	177.7±179.2(12)	0.1253	Vagus mn, semi-intact prep.	2 C,G
Width 50 RRSI (ms)	1075.3±810.7(10)	591.1±409.5(12)	0.1105	Vagus mn, semi-intact prep.	2 D, H
Amplitude RRSI (pA)	374.6±210.7(10)	260.1±158.8(12)	0.1616	Vagus mn, semi-intact prep.	2 E, I
Firing frequency RRSI (Hz)	54.4±23.4(8)	48.7±34.5(6)	0.7171	Vagus mn, semi-intact prep.	3 C, G
Number of spikes per RRSI	89.6±83.1(8)	48.7±28.1(6)	0.2276	Vagus mn, semi-intact prep.	3 D, H
Action Potential threshold (mV)	-45.3±2.8(8)	-47.9±3.8(6)	0.1627	Vagus mn, semi-intact prep.	3 E, I
Membrane potential (mV)	-54.2±4.3(10)	-57.5±9.8(12)	0.3013	Vagus mn, semi-intact prep.	4 C, F
Max Firing frequency (Hz)	49.1±23.5(10)	48.7±34.5(12)	0.9780	Vagus mn, semi-intact prep.	4 D, G
Membrane potential (mV)	-51.0±5.1(8)	-49.5±6.9(10)	0.6296	LC neuron, slice	5 A, F
Firing frequency (Hz)	32.8±10.9(8)	51.0±35.3(10)	0.1528	LC neuron, slice	5 B, G
Membrane potential (mV)	-55.3±10.8(8)	-50.8±5.9(8)	0.3205	Pallium neuron, slice	5 D, H
Firing frequency (Hz)	20.2±11.5(8)	23.0±6.1(8)	0.5599	Pallium neuron, slice	5 E, I

Data are the mean±SEM(*n*). Statistical significance was determined by unpaired t test. The values were extracted from baseline values reported in the figures indicated in the last column
