# Supplementary material for: Synaptic modifications transform neural networks to function without oxygen
Source: BMC Biol. 2023 Mar 16;21:54. doi: 10.1186/s12915-023-01518-0 (PMC10022038; doi:10.1186/s12915-023-01518-0)
Supplement: Supplementary file 2 — Additional file 2: Figure S1. Respiratory-related synaptic input recorded in an overwintered motoneuron for 2 hours in hypoxia. [file 12915_2023_1518_MOESM2_ESM.pdf]

SUPPLEMENTAL FIGURES

FIGURE S1

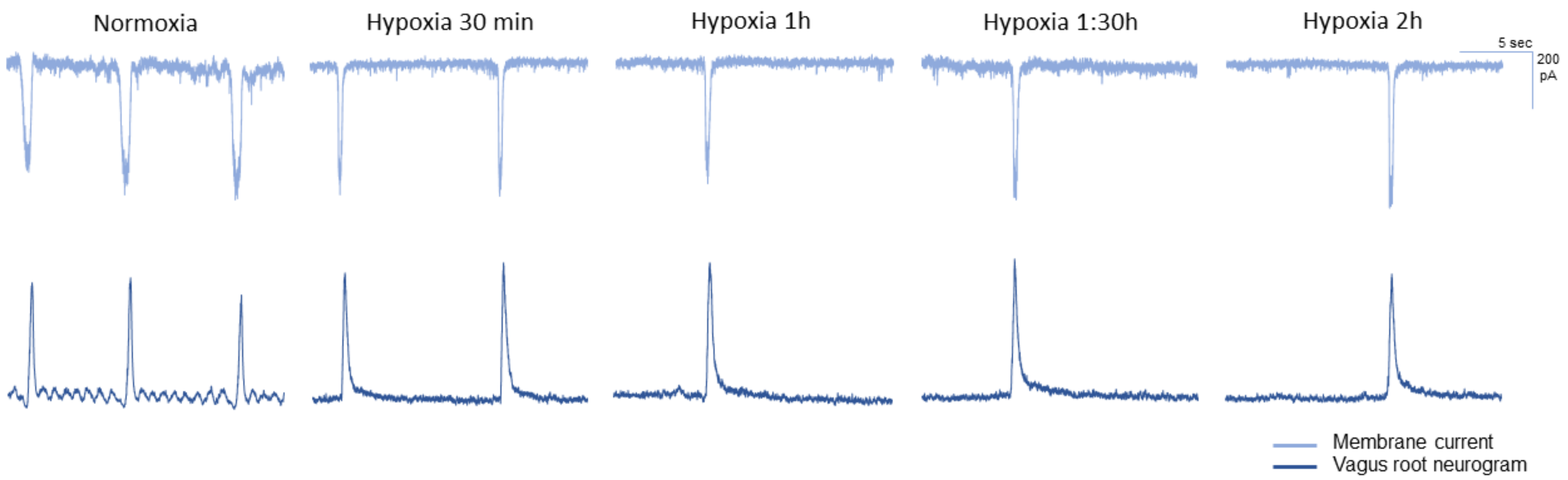

Additional file 2: Figure S1. Respiratory-related synaptic input recorded in an overwintered motoneuron for 2 hours in hypoxia. Top- Recording of the motoneuron membrane current. Bottom – concurrent recording of the network output.
