# Supplementary material for: Synaptic modifications transform neural networks to function without oxygen
Source: BMC Biol. 2023 Mar 16;21:54. doi: 10.1186/s12915-023-01518-0 (PMC10022038; doi:10.1186/s12915-023-01518-0)
Supplement: Supplementary file 3 — Additional file 3: Figure S2. Concurrent recording of vagal motoneuron action potentials and respiratory network output of a control frog during hypoxia-induced network failure. [file 12915_2023_1518_MOESM3_ESM.pdf]

FIGURE S2

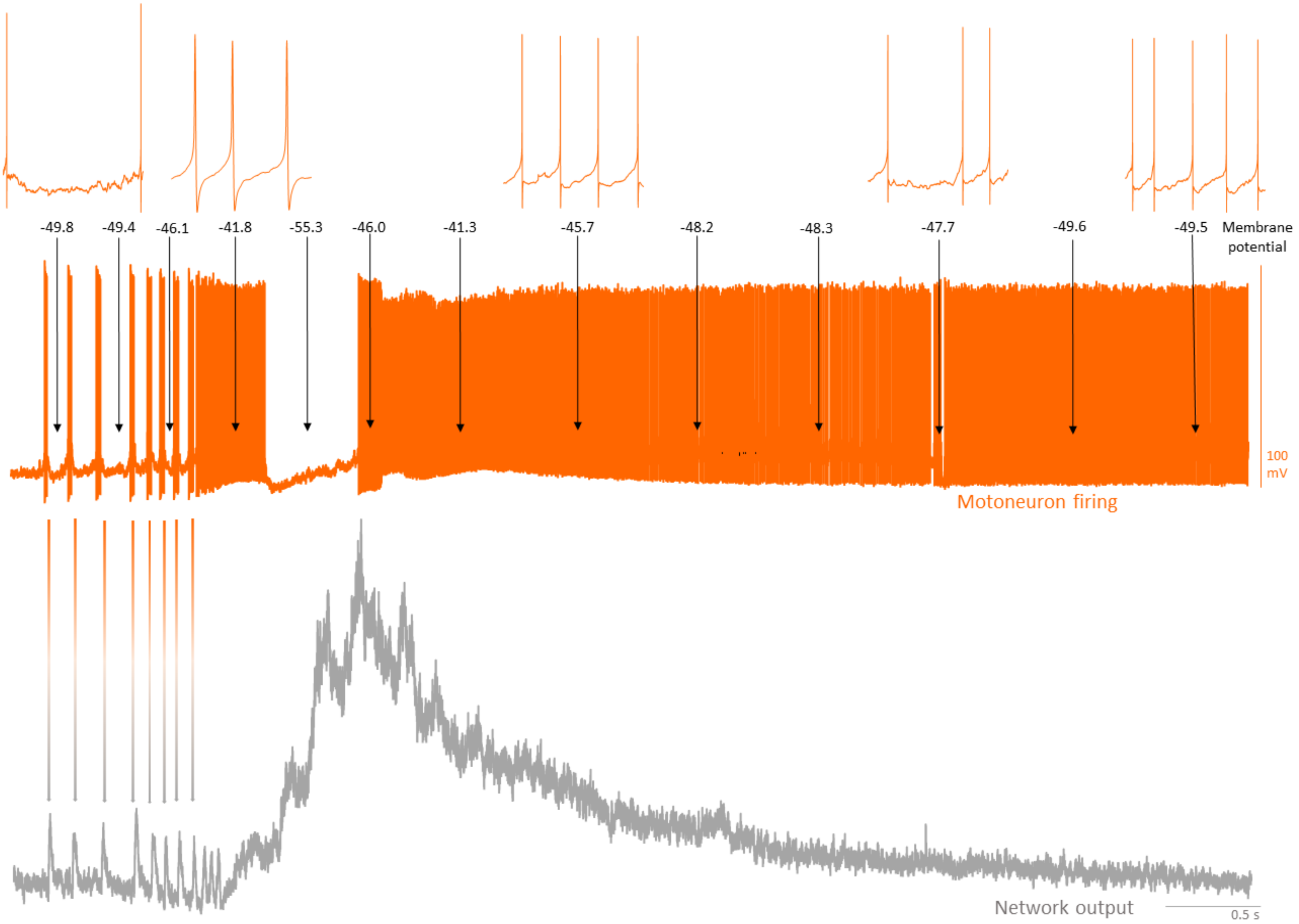

Additional file 3: Figure S2. Concurrent recording of vagal motoneuron action potentials and respiratory network output during hypoxia-induced network failure. The firing of the control frog motoneuron was synchronous to the network output recorded in the vagal root (bottom) until the failure of the network. After network failure, the motoneuron proceeds to fire tonically. Membrane potential was analyzed throughout the recording in the sites indicated with a black arrow, and details of the membrane current are shown on the top.
