# Supplementary material for: Synaptic modifications transform neural networks to function without oxygen
Source: BMC Biol. 2023 Mar 16;21:54. doi: 10.1186/s12915-023-01518-0 (PMC10022038; doi:10.1186/s12915-023-01518-0)
Supplement: Supplementary file 4 — Additional file 4: Figure S3. Recording of a control vagal motoneuron firing in response to a 1000pA step current in baseline conditions and after 10 minutes of network failure using a pipette solution with no ATP. [file 12915_2023_1518_MOESM4_ESM.pdf]

FIGURE S3

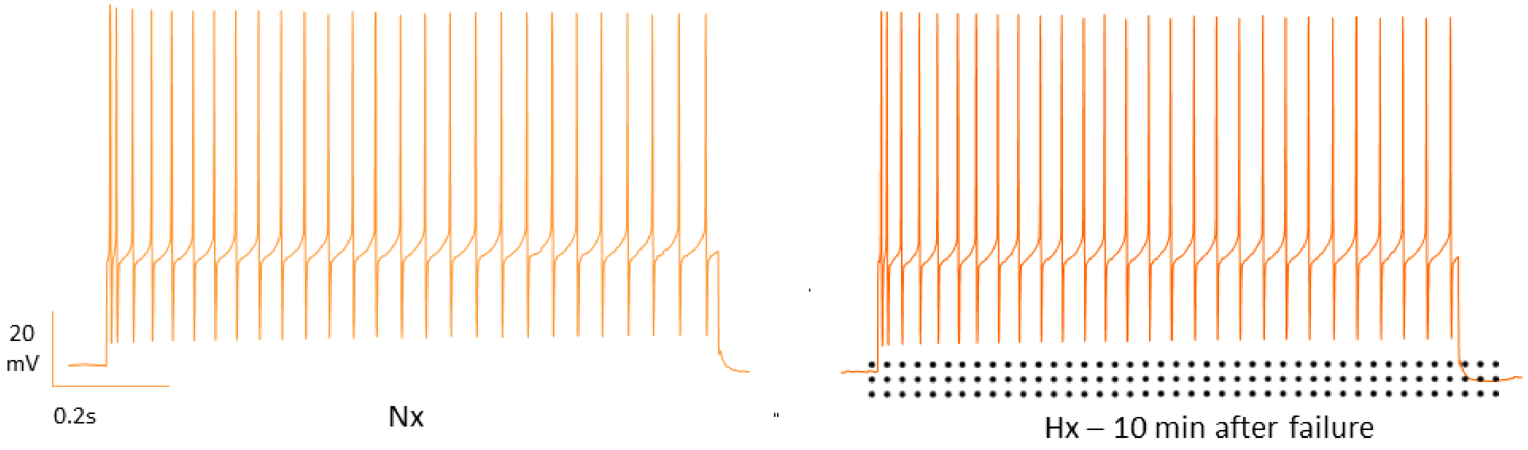

Additional file 4: Figure S3. Recording of a control vagal motoneuron patched using a pipette solution with no ATP. Firing in response to a 1000pA step current was measured in baseline conditions (left) and after 10 minutes after network failure (right).
