# Supplementary material for: Synaptic modifications transform neural networks to function without oxygen
Source: BMC Biol. 2023 Mar 16;21:54. doi: 10.1186/s12915-023-01518-0 (PMC10022038; doi:10.1186/s12915-023-01518-0)
Supplement: Supplementary file 5 — Additional file 5: Figure S4. Recording of a control motoneuron showing that it does not change firing in response to intermittent current injection up to 1 hour of hypoxia exposure. [file 12915_2023_1518_MOESM5_ESM.pdf]

FIGURE S4

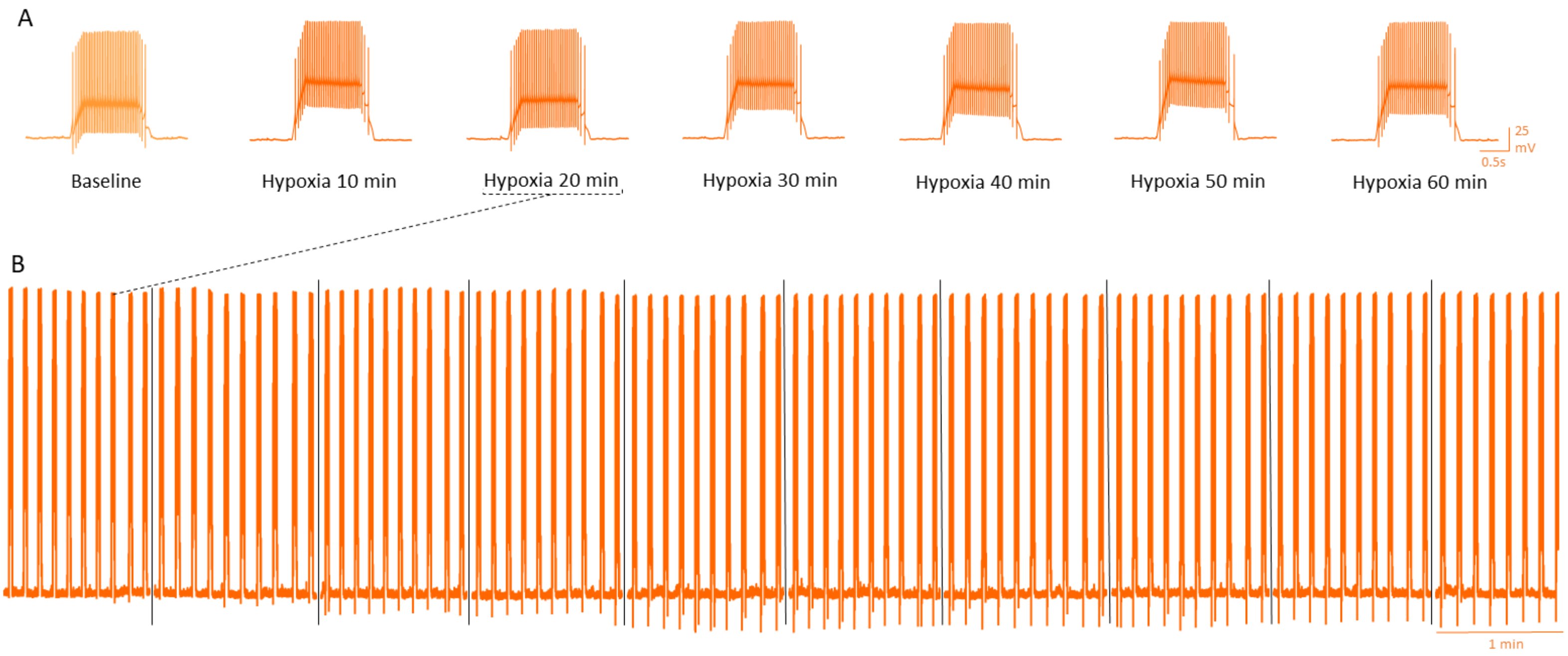

Additional file 5: Figure S4. Control motoneuron does not change firing in response to intermittent current injection in hypoxia. (A) Detail of representative traces showing firing in response to a 1000pA step current in baseline conditions (left) and throughout one hour of hypoxia (right). This current was injected every 5 seconds, as shown in ten minutes of recording in (B).
